# Extracellular cell-free RNA profile in human large follicles and small follicles

**DOI:** 10.3389/fcell.2022.940336

**Published:** 2022-09-26

**Authors:** Huajuan Shi, Min Pan, Yuqi Sheng, Erteng Jia, Ying Wang, Juan Dong, Jing Tu, Yunfei Bai, Lingbo Cai, Qinyu Ge

**Affiliations:** ^1^ State Key Laboratory of Bioelectronics, School of Biological Science and Medical Engineering, Southeast University, Nanjing, China; ^2^ School of Medicine, Southeast University, Nanjing, China; ^3^ Clinical Center of Reproductive Medicine, State Key Laboratory of Reproductive Medicine, First Affiliated Hospital, Nanjing Medical University, Nanjing, China

**Keywords:** 5890, figures: 7, supplemental figures: 4, supplemental tables: 4, cell-free RNA, follicular fluid, RNA-seq, extracellular

## Abstract

**Background:** Previous studies have shown that a large number of valuable and functional cell-free RNAs (cfRNAs) were found in follicular fluid. However, the species and characteristics of follicular fluid cfRNAs have not been reported. Furthermore, their implications are still barely understood in the evaluation of follicular fluid from follicles of different sizes, which warrants further studies.

**Objective:** This study investigated the landscape and characteristics of follicular fluid cfRNAs, the source of organization, and the potential for distinguishing between follicles of different sizes.

**Methods:** Twenty-four follicular fluid samples were collected from 20 patients who received *in vitro* fertilization (n = 9) or ICSI (n = 11), including 16 large follicular fluid and 8 small follicular fluid samples. Also, the cfRNA profile of follicular fluid samples was analyzed by RNA sequencing.

**Results:** This result indicated that the concentration of follicular fluid cfRNAs ranged from 0.78 to 8.76 ng/ml, and fragment length was 20–200 nucleotides. The concentration and fragment length of large follicular fluid and small follicular fluid samples were not significantly different (*p* > 0.05). The technical replica correlation of follicular fluid samples ranged from 0.3 to 0.9, and the correlation of small follicular fluid samples was remarkably (*p* < 0.001) lower than that of large follicular fluid samples. Moreover, this study found that cfRNAs of the follicular fluid could be divided into 37 Ensembl RNA biotypes, and a large number of mRNAs, circRNAs, and lncRNAs were observed in the follicular fluid. The number of cfRNAs in large follicular fluid was remarkably (*p* < 0.05) higher than that of small follicular fluid. Furthermore, the follicular fluid contained a large amount of intact mRNA and splice junctions and a large number of tissue-derived RNAs, which are at a balanced state of supply and elimination in the follicular fluid. KEGG pathway analysis showed that differentially expressed cfRNAs were enriched in several pathways, including thyroid hormone synthesis, the cGMP-PKG signaling pathway, and inflammatory mediator regulation of TRP channels. In addition, we further showed that four cfRNAs (TK2, AHDC1, PHF21A, and TTYH1) serve as a potential indicator to distinguish the follicles of different sizes. The ROC curve shows great potential to predict follicular fluid from follicles of different sizes [area under the curve (AUC) > 0.88].

**Conclusion:** Overall, our study revealed that a large number of cfRNAs could be detected in follicular fluid and could serve as a potential non-invasive biomarker in distinguishing between follicles of different sizes. These results may inform the study of the utility and implementation of cfRNAs in clinical practice.

## Background

The World Health Organization (WHO) predicts that infertility will become the third most serious disease worldwide in the 21st century, with an average incidence of 9% among couples of reproductive age (Boivin et al., 2007; Inhorn and Patrizio, 2015; Kushnir et al., 2017). During the past 20 years, assisted reproductive technology (ART) has gradually entered the public view with the development of reproductive medicine, and about 2% of infertility patients are conceived through using ART (Yang et al., 2014; De Geyter, 2019). Although there have been major advances in ART, the evaluation of embryo potential is still limited, which may increase pregnancy failure and multiple pregnancies (Braga et al., 2016). At the present, with the increasing use of oocyte and ovarian cryopreservation in elderly women or women with premature ovarian failure, it is becoming more and more important to accurately evaluate the quality of the oocyte. The embryos with the highest implantation potential rely only on morphological criteria at present. Nevertheless, it is not a powerful and reliable indicator to evaluate the quality of oocytes and their developmental ability by using morphological criteria (Scalici et al., 2014). Additionally, the current genetic screening methods for embryos are invasive. For example, preimplantation genetic testing (PGT) methods based on oocyte polar body biopsy, blastomere of cleavage embryos, and blastocyst trophoblast ectoderm (TE) cells have been shown to produce acceptable pregnancy results (De Rycke et al., 2017). But the invasive nature of these methods poses a potential threat to subsequent biopsy embryo development. Hence, many studies have focused on the identification of non-invasive methods based on oocyte microenvironment analysis to improve the accuracy of embryo selection (Wang and Sun 2006; Patrizio et al., 2007; Zhang et al., 2021).

Previous studies have shown that the components of follicular fluid (FF), secreted by granulosa cells and theca cells and diffused by capillaries, were investigated as potential biomarkers for predicting the quality of oocytes and embryos (Da Broi et al., 2018; Montani et al., 2019; Huang et al., 2021; Lazzarino et al., 2021). Actually, follicular fluid is a unique biological fluid, which is a complex mixture of ions, proteins, and metabolite compounds reflecting follicular metabolism (Hanrieder et al., 2008; Hanrieder et al., 2009). In addition, studies have shown that nutrients and metabolites in follicular fluid are closely related to reproductive diseases (Kim et al., 2006), oocyte quality (Berker et al., 2009), embryo quality, and the outcome of *in vitro* fertilization (IVF) attempts (Wu et al., 2007). Therefore, follicular fluid may be a reliable source of biomarkers of oocyte and embryonic outcomes and can be used as prognostic/diagnostic tools in ART (Cai et al., 2019; Huo et al., 2020; Qasemi and Amidi 2020; Wang J et al., 2021).

Recent studies have showed that a large number of cell-free RNAs (cfRNAs) are observed in follicular fluid. Among them, microRNAs (miRNAs) have been studied extensively, which could be used to distinguish high-quality embryos from low-quality embryos and affect ovarian function and follicular development, and as non-invasive tools for the prediction of blastulation (Fu et al., 2018; Li X et al., 2019). However, the number of miRNAs in follicular fluid is relatively less and non-specific, which limits their extensive application. Furthermore, studies have shown that long RNA species, including circular RNAs (circRNAs) and long noncoding RNAs (lncRNAs), have also been found in human follicular fluid, and their expression changes may be related to reproductive disorders (Santonocito et al., 2014; Jiao et al., 2018). In other words, lncRNAs can be highly enriched in the follicular fluid of mature follicles of polycystic ovary syndrome (PCOS) (Jiao, Shi, Wang, Fang, Cao, Zhou, Wang and Li, 2018). These results indicated that follicular fluid contains a large amount of valuable and functional cfRNAs. However, to date, no studies have reported the variety and characteristics of cfRNAs in follicular fluid. In addition, their implications in evaluating follicles’ quality and growth environment are still barely understood, which warrants further studies.

Hence, the present study was conducted to observe the following: 1) the characterization of follicular fluid cfRNAs was first carried out, and its existence pattern and regularity were analyzed. The potential to distinguish between oocytes of different sizes was further studied; 2) the complexity of follicular fluid cfRNA transcripts was analyzed, which includes 37 Ensembl RNA biotypes. Also, a large number of mRNAs, circRNAs, and lncRNAs were observed in the follicular fluid; 3) the integrity of follicular fluid mRNA was analyzed. This result showed that follicular fluid contains a large number of intact mRNAs and spliced junctions; 4) the analysis of tissue-specific gene sources in follicular fluid. This result indicated the presence of RNAs derived from tissue-specific genes in follicular fluid; 5) follicular fluid cfRNAs can be used as a potential indicator to distinguish the quality of follicles. Overall, this study revealed the types of follicular fluid cfRNAs and fragment distribution, source of organization, and the potential to predict different follicle sizes. These results may inform the study of the utility and implementation of cfRNAs in clinical practice.

## Materials and methods

### Ethical statement

All patients participating in this study provided written informed consent. Ovarian follicular fluid was collected at the Clinical Center of Reproductive Medicine in the First Affiliated Hospital, Nanjing, China. The study was approved by the Ethics Committee of the First Affiliated Hospital of Nanjing Medical University (Jiangsu Province Hospital; No: 2021-SR428) and conducted in accordance with the Declaration of Helsinki.

### Clinical characteristics of patients

In this study, samples were collected from 20 patients who received IVF (n = 9) or ICSI (n = 11) cycles at the Clinical Center of Reproductive Medicine in the First Affiliated Hospital, Nanjing, China. The clinical features of patients are shown in Supplementary Table S1. The patients’ ages ranged from 27 to 43 (32.27 ± 5.78 years), and body mass index (BMI) ranged from 18 to 25.4 kg/m^2^ (22.1 ± 2.29 kg/m^2^). Except for two patients with ovarian insufficiency, the ovarian reserve was normal in 80.9% of patients. Anti-Mullerian hormone (AMH) was between 1.03 and 10.33 (4.72 ± 3.36). The baseline hormonal levels, including FSH, LH, and 17 β estradiol (E2), were assessed on day 3 of the menstrual cycle.

### *In vitro* fertilization

A total of 30 follicular fluid samples were obtained from 20 patients (a mean of 1.5 ± 1.1 follicular fluid samples can be collected per patient). The follicular growth was evaluated by measuring transvaginal ultrasound and serum 17 β-estradiol (E_2_) concentration. When at least three follicles were 18 mm or more in diameter, injection of human chorionic gonadotrophin (hCG) promotes ovulation. Then, 36 h after hCG injection, the follicles larger than 10 mm in diameter were aspirated by ultrasound guidance. Among them, follicles greater than 18 mm in diameter were considered large follicles, and follicles of 10–18 mm in diameter were considered small follicles. To minimize maternal contamination, the cumulus cells of oocytes surrounding them should be removed as much as possible before ICSI or IVF cycles.

### Follicular fluid collection and cell-free RNA isolation

Only clear follicular fluid samples were included; blood-stained and cloudy follicular fluid samples were excluded to avoid contamination. Thereafter, the samples of follicular fluid were centrifuged at 12000 × g for 10 min to remove cell fragments and then immediately stored at −80°C for standby application. TRIzol^®^ reagent (Thermo Fisher Scientific, #1559608) was used, according to the manufacturer’s instructions, to extract total RNA from follicular fluid samples within 4 h and was treated with RQ1 RNase-free DNase (TaKaRa Co. Ltd., Japan) to eliminate genomic DNA contamination. Then, the quality and quantity of total RNA were measured using an Agilent 2100 Bioanalyzer (Agilent Technologies, Palo Alto, CA, United States). The OD260/OD280 ratio was used as the RNA purity index. If the ratio of OD260/OD280 is 1.8–2.1, the RNA purity is acceptable. Its integrity was further determined by 1.5% formaldehyde denaturing agarose gel electrophoresis. Then, total RNA was stored at −80°C until further use.

### Library preparation and sequencing

We used SMARTer-seq® Stranded Kit User Manual (TaKaRa Bio. Inc., Japan) and modified some procedures to prepare the RNA-seq library. Briefly, 1) the total RNA was treated with DNase I (NEB) for 15 min at 37 °C and purified by VAHTS^*@*^ RNA Clean Beads (Vazyme Biotech Co., Ltd.). 2) The purified RNAs are fragmented at 85°C for 2 min. 3) Then, 10 cycles were used for the first round of PCR to add Illumina Adapters and Indexes (PCR products could be stored at −20°C for up to 2 weeks). 4) Depletion of ribosomal cDNA with ZapR and R-Probes. 5) The final RNA-seq library was amplified by using 16 cycles. 6) The final RNA-seq library was purified by using VAHTS^*@*^ DNA Clean Beads (Vazyme Biotech Co. Ltd.). 7) The cDNA library concentration was measured using a Qubit^®^ 2.0 Fluorometer (>1 ng/μl). The constructed library was sequenced by using the Illumina HiSeq 2500 (Illumina Inc., San Diego, CA, United States) sequencer (2 × 150-bp paired-end pattern (PE150)).

### Data filtering and quality control

Prior to sequencing data mapping, raw reads (raw data) with adapters, > 5% unknown nucleotides, and low-quality reads containing >20% of bases with qualities of <20 were removed. The results of quality control are shown in Supplementary Table S2. All downstream analyses used high-quality filtered data.

### RNA-seq analysis

The raw sequencing reads were filtered by FastQC (v0.11.4) and aligned using the spliced read aligner HISAT2 (version 2.1.0) (Kim et al., 2015), which was supplied with UCSC hg19 as the reference genome with GENCODE v35 transcript information. The expression levels of each transcript were normalized by quantifying FPKM (fragments per kilobase of exon model per million mapped fragments). Annotations of mRNA and lncRNA in the human genome were retrieved from the GENCODE (v25) database. The bedtools coverage tool was used to get the coverage of exons, which were obtained from the GENCODE (V25) database. Annotations of different regions (UTR and CDS) were also obtained from the UCSC Table Browser, and bedtools multicov was performed to analyze read counts in different regions.

For the first time, clean paired-end RNA-seq reads were mapped to the human reference genome (UCSC hg19) with BWA (Li and Durbin, 2009). The circRNA analysis was achieved using the CIRI2 tool (Gao et al., 2015). Differentially regulated RNAs were annotated with gene IDs and assessed for Kyoto Encyclopedia of Genes and Genomes (KEGG) pathway enrichment using DAVID (https://david. ncifcrf.gov/).

### Data and statistical analyses

The RNA-seq raw read counts were normalized by FPKM. Variates with a frequency of <25% (i.e., less than 25% expressed throughout the sample) were omitted, and the remaining transcripts were used for subsequent analyses. The identified genes were analyzed for differential expression by the Mann–Whitney U test in the large follicular fluid and small follicular fluid samples, and the *p*-value of each transcript was adjusted by the Benjamini–Hochberg method to control the false discovery rate (FDR). The genes of FDR < 0.01 and fold change > 2 were retained. Only RNAs upregulated in these follicular fluids (FDR < 0.01, fold change > 2) were considered valuable for oocyte classification.

All statistical analyses were student’s t-tests, and the *p*-value <0.05 was considered statistically significant. Figures were created by GraphPad Prism 7.0 (GraphPad Software, Inc. La Jolla, CA, United States) and R software package.

## Results

### Concentration and size distribution of cell-free RNAs in human follicular fluid

**FIGURE 1:**
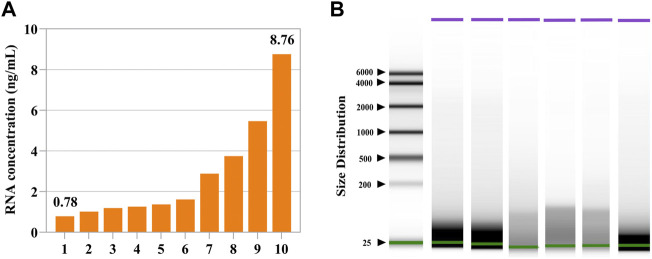
Concentrations and fragments of cell-free RNAs in the human follicular fluid. **(A)** Measured concentrations of cell-free RNAs in ten follicular fluids. **(B)** Fragment distributions of cell-free RNAs in six follicular fluids. Nearly all detected cell-free RNAs were within the size range of 20–200 nt.

As shown in Figure 1, the concentrations of follicular fluid cfRNAs ranged from 0.78 ng/ml to 8.76 ng/ml (Figure 1A). Nearly all detected cfRNA fragment lengths ranged from 20 to 200 nucleotides (nt) (Figure 1B).

In terms of follicular fluids of different-sized follicles, the RNA concentrations ranged from 0.2 ng/ml to 10.2 ng/ml and 1.36 ng/ml to 7.32 ng/ml in large follicular fluid and small follicular fluid samples, respectively (Supplementary Figure S1A). There was no significant (*p* > 0.05) difference in the distribution range of cfRNA fragments in follicular fluids of different-sized follicles, but small-sized follicular fluid fragments were more diffused (Supplementary Figure S1B,S1C).

### Quality control and the reads distribution fragments of cell-free RNAs in human follicular fluid

**FIGURE 2:**
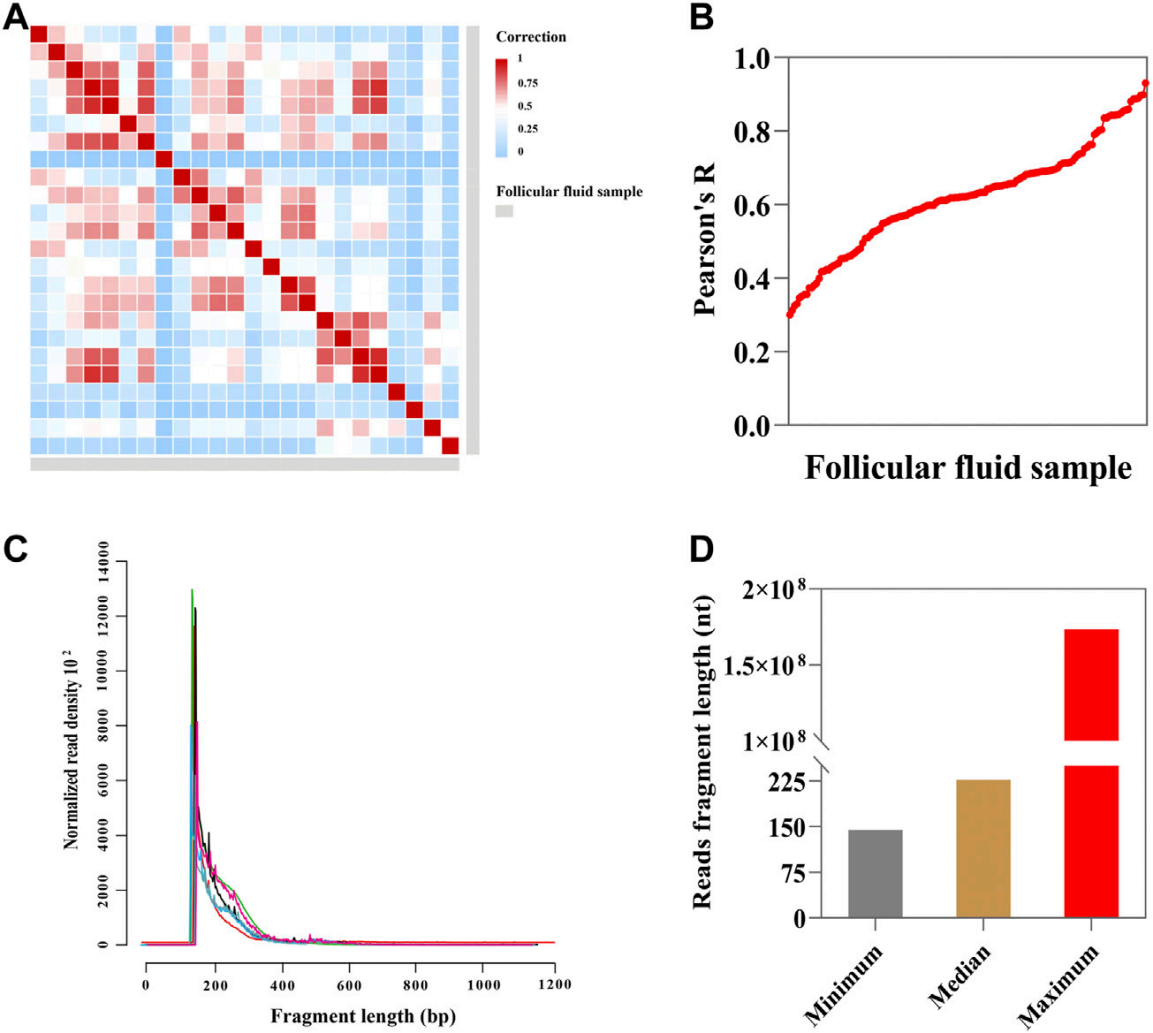
Sequencing and descriptive characteristics of reads. **(A)** Heatmap showing Pearson correlation of FPKM (fragments per kilobase of exon model per million mapped fragments) values (n = 24; blue indicates low correlation and red indicates high correlation); **(B)** correlation between each follicular fluid sample (n = 24, Pearson correlation analysis). **(C)** Fragment size distribution of sequence reads in follicular fluid (n = 7). **(D)** Minimum, median, and maximum read fragment sizes of the follicular fluid (n = 24).

We generated 24 total RNA-seq datasets from 24 follicular fluids and obtained a total of ∼27 billion reads (∼1.1 billion reads per sample, Supplementary Figure S2A,S2B and Supplementary Table S2). Additionally, we checked the correlation between two technical replicas. This result shows that the technical replica correlation of follicular fluid samples ranged from 0.3 to 0.9 (Pearson correlation analysis) (Figures 2A,B). The read fragment distribution of follicular fluid cfRNAs was mainly concentrated at about 200 bp (Figure 2C). The minimum, median, and maximum read fragment lengths of follicular fluid cell-free RNAs were 144, 226, and 173361750, respectively (Figure 2D).

As shown in Supplementary Figure S2C, the correlation of follicular fluid between two technical replicates indicated high repeatability (*R* ^*2*^ = 0.888). The small follicular fluid sample profile showed a poor correlation between two technical replicas (*R* ^*2*^ = 0.5106, Supplementary Figure S2D). The correlation of small follicular fluid samples was significantly (*p* < 0.001) lower than that of large follicular fluid samples, with the median of *R* ^*2*^ = 0.6515 and 0.418 in large follicular fluid and small follicular fluid samples, respectively (Supplementary Figure S2E). In addition, as can be seen from Supplementary Figure S2F, the read fragment distribution of cfRNAs was mainly concentrated at about 200 bp in large follicular fluid samples. The read fragments of cfRNAs ranged from 200 bp to 1,200 bp or even longer in small follicular fluid samples and without obvious regularity (Supplementary Figure S2G). Nevertheless, there was no statistical significance in the mean length of follicular fluid read fragments between the two groups (*p* > 0.05) (Supplementary Figure S2H).

### Relative abundance and complexity of cell-free RNA transcripts in human follicular fluid

**FIGURE 3:**
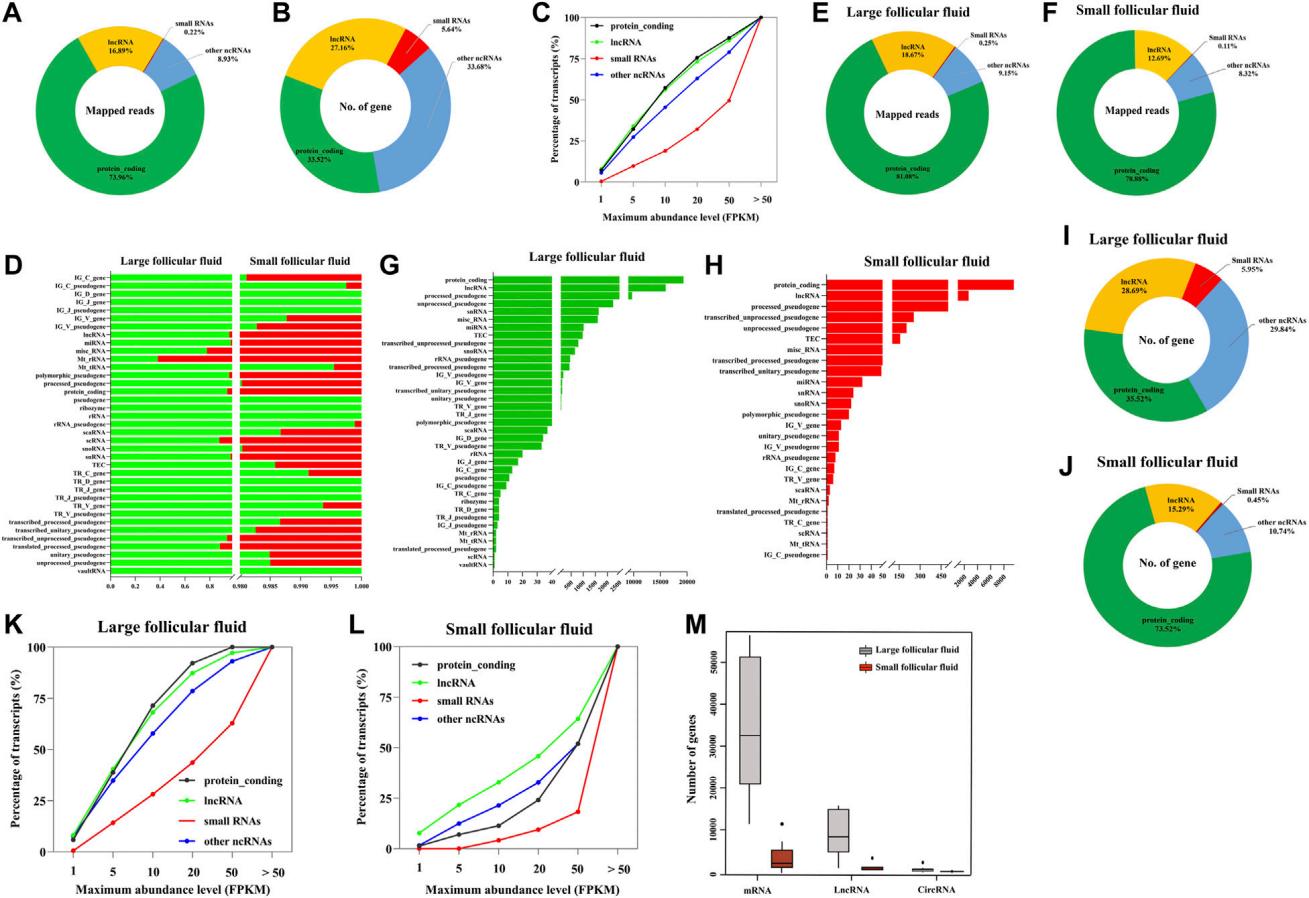
Biotype analysis of RNA was identified in follicular fluid. **(A)** Proportions mapped reads to various types of RNA. **(B)** Distribution of genes in follicular fluid. **(C)** RNAs are plotted as the percentage of quantified transcripts against the expression level in follicular fluid. **(D)** Percentage of total aligned reads for RNA in follicular fluid by Ensembl biotypes. Numerical values for each biotype are found in Supplementary Table S3. **(E)** and **(F)** Distribution of mapped reads to the annotated genes in large follicular fluid and small follicular fluid samples, respectively. **(G)** and **(H)** Distribution of genes in large follicular fluid and small follicular fluid samples by Ensembl biotypes, respectively; **(I)** and **(J)** Distribution of genes in large follicular fluid and small follicular fluid samples, respectively. **(K)** and **(L)** RNAs are plotted as the percentage of quantified transcripts against the expression level in large follicular fluid and small follicular fluid samples, respectively. **(M)** Distribution of RNAs per sample among large follicular fluid (n = 16) and small follicular fluid (n = 8).

We analyzed the cfRNAs of follicular fluid, and a large majority of the mapped reads corresponded to protein_coding RNAs and lncRNAs, respectively (Figure 3A). Most of the identified genes were protein_coding genes, lncRNAs, and other noncoding RNAs (other ncRNAs) (Figure 3B). In addition, we found that the majority of annotated protein_coding genes, lncRNA, small RNAs, and other ncRNAs were expressed very highly in follicular fluid (> 1 FPKM) (Figure 3C). The most abundant RNA was RN7SL5P, which had an average expression level of 18861 FPKM across all samples.

Furthermore, in order to detail the analysis of the biological types of follicular fluid cfRNAs of different sizes, we analyzed the number of follicular fluid cfRNA-aligned reads, which were classified into 37 Ensembl RNA biotypes (Supplementary Table S3). In the removal gene expression biotypes of a single expression level (such as IG_D_gene, IG_J_gene, and IG_J_pseudogene) (Figure 3D), we found the differences in the total aligned reads of each biotype between large follicular fluid and small follicular fluid samples. The significant (*p* < 0.05) changes were observed in the sequenced read counts of protein_coding, lncRNA, and miRNA (Supplementary Table S3). Mt_rRNA and Mt_tRNA showed the higher aligned reads in large follicular fluid samples than in small follicular fluid samples but with no significant difference (*p* > 0.05) (Supplementary Table S3). Furthermore, in order to better understand the biotype distribution of follicular fluid cfRNAs, we visualized this using a ring chart (Figures 3E,F). This result showed that protein_coding and lncRNA accounted for the majority of RNAs (91.29% of large follicular fluid and 91.57% of small follicular fluid).

As can be seen from Figures 3G,H, the biotype and number of genes in large follicular fluid samples were higher than those of small follicular fluid samples. The number of protein_coding genes and lncRNA accounted for 57.16 and 88.24% of the total number of genes in large follicular fluid and small follicular fluid samples, respectively (Figures 3I,J). In addition, we observed that the majority of annotated protein_coding genes, lncRNA, small RNAs, and other ncRNAs were expressed very highly in follicular fluid having > 1 FPKM (Figure 3K,L **,** Supplementary Table S4)**.** In large follicular fluid samples, the expression levels of protein_coding genes and lncRNAs were higher than those of small RNAs and other ncRNA expression levels. In contrast, the expression level of lncRNAs in small follicular fluid samples was slightly higher than that of the other three RNAs. For large follicular fluid samples, we detected 40903 mRNAs, 360 circRNAs, and 11560 lncRNAs in the median (Figure 3M). In a small follicular fluid sample, we detected 2,391 mRNAs, 13 circRNAs, and 207 lncRNAs in the median (Figure 3M)**.**

### Circular RNAs are abundant and enriched in follicular fluid

**FIGURE 4:**
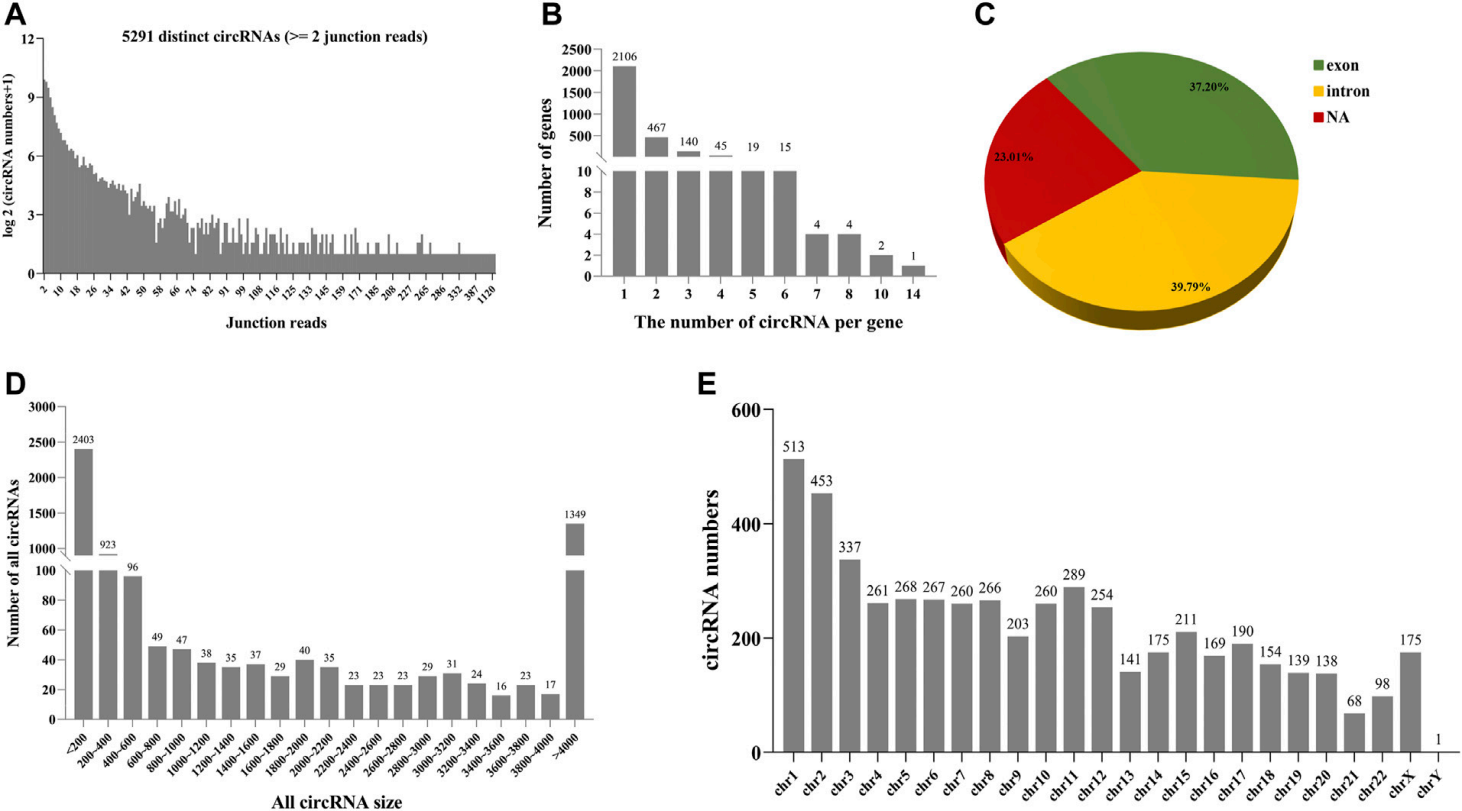
Number and characteristics of identified circRNAs in follicular fluid. **(A)** Number of identified circRNAs and junction reads in follicular fluid. **(B)** Number distribution of formed circRNAs per gene in follicular fluid. **(C)** Source of circRNAs identified in follicular fluid. NA: not assigned. **(D)** Distribution of circRNA length in follicular fluid. **(E)** Distribution of circRNAs in different chromosomes.

As can be seen from Figure 4, 5291 distinct candidates of circRNAs were found in follicular fluid, including at least two unique back-spliced reads (Figure 4A). Here, 75% of host genes produced only one circRNA (Figure 4B). Of note, > 2 circRNAs were generated from certain genes. About 77% of the circRNAs overlapped with known genes in follicular fluid (Figure 4C). Among them, the percentage of exon circRNAs was 37.2% in follicular fluid. In addition, the lengths of most 74.5% circRNAs ranged between 200 and 4,000 nucleotides, and most of them are located in chromosome 1 in follicular fluid (Figures 4D,E).

In addition, as shown in Supplementary Figures S3A,B, 5248 and 89 different candidate circRNAs were found in large follicular fluid and small follicular fluid samples, including at least two unique back-spliced reads. About 76.9 and 93.1% of the circRNAs overlapped with known genes in large follicular fluid and small follicular fluid samples, respectively (Supplementary Figures S3C,D). Among them, the percentages of exon circRNAs were 36.82 and 86.21% in large follicular fluid and small follicular fluid, respectively. The lengths of most 74.7 and 42.1% circRNAs ranged between 200 and 4,000 nucleotides in large follicular fluid and small follicular fluid samples, respectively (Supplementary Figure S3E), and most of them were located in chromosome 1 in large follicular fluid and small follicular fluid (Supplementary Figure S3F).

### Follicular fluid contain a substantial fraction of intact mRNAs and a large number of spliced junctions

**FIGURE 5:**
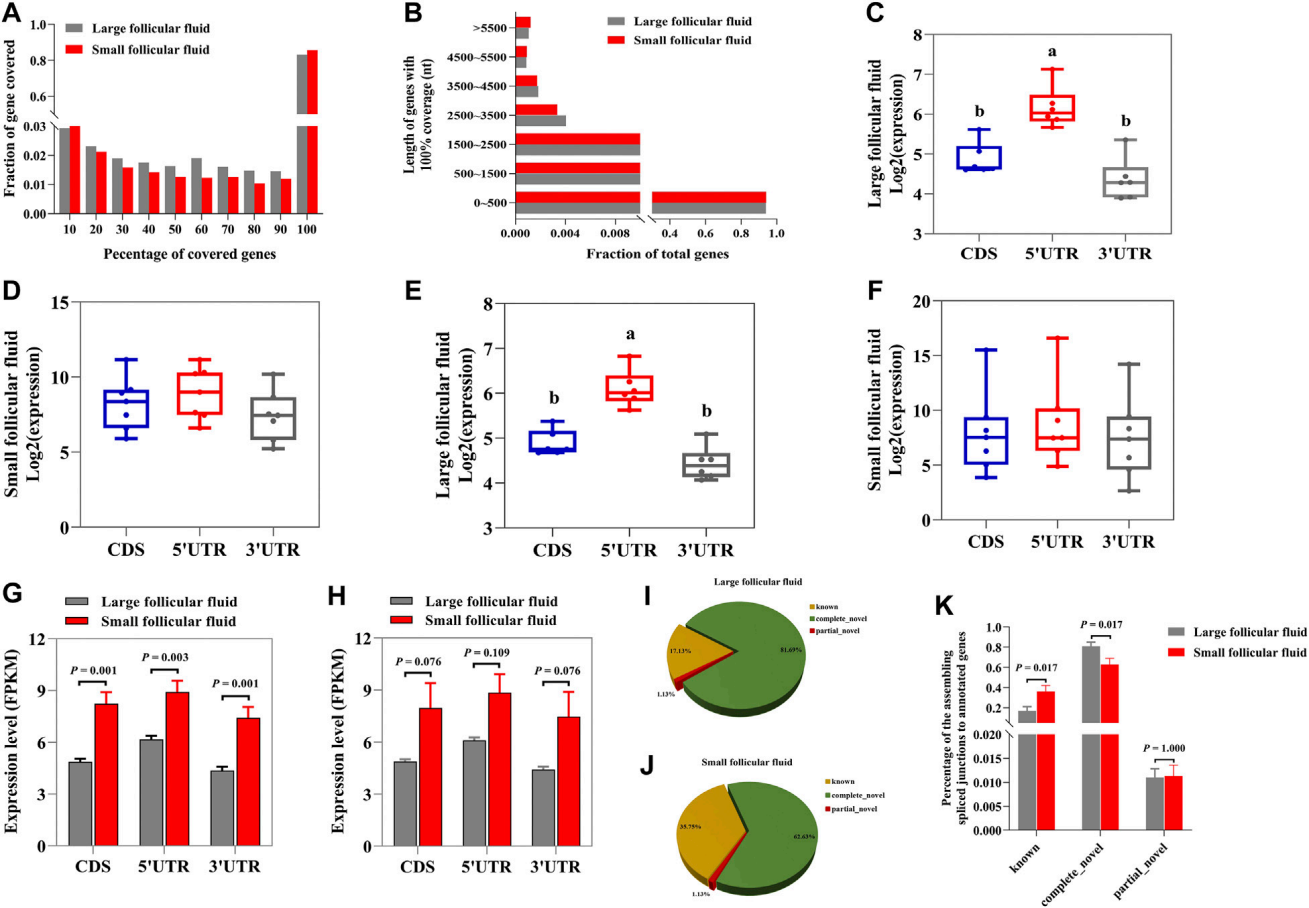
Follicular fluid contains quite a portion of intact mRNAs and a large number of spliced junctions. **(A)** Percentage of gene coverage for large follicular fluid and small follicular fluid. **(B)** Length distribution of intact mRNAs in large follicular fluid and small follicular fluid samples. The relative expression levels of the UTR and CDS regions of full length in large follicular fluid **(C)** and small follicular fluid samples **(D)**. **(E)** and **(F)** are the relative expression levels of the UTR and CDS regions of the other transcripts. **(G)** Statistical analysis of CDS and UTR regions in large follicular fluid and small follicular fluid for the full-length transcripts. **(H)** Statistical analysis of CDS and UTR regions in large follicular fluid and small follicular fluid samples for the other transcripts. **(I)** and **(J)** Distribution of the spliced junctions to annotated genes in large follicular fluid and small follicular fluid samples, respectively. **(K)** Percentage of the spliced junctions to annotated genes in large follicular fluid and small follicular fluid. Bars assigned with different letters are remarkably different (*p* < 0.05). ***p < 0.001, **p < 0.01, and *p < 0.05.

Next, we studied whether the identified mRNAs in follicular fluid were intact or fragmented. We calculated the coverage of protein_coding genes by using mapped reads. In large follicular fluid and small follicular fluid samples, 83.15 and 85.68% of reads were across full-length genes (100% coverage), respectively (Figure 5A). The length of intact mRNAs in large follicular fluid samples varied from 9 to 205,012 nt, with an average of 2,568 nt, but it ranged from 30 to 347,778 nt, with an average of 5,363 nt in small follicular fluid samples (Figure 5B)**.** Then, the full-length mRNA reads were aligned with coding sequences (CDSs), 5′-untranslated (5′UTR), and 3′-untranslated (3′UTR) regions. The result showed that 5′UTR regions in large follicular fluid samples were remarkably higher (*p* < 0.05) than CDS and 3′UTR regions (Figure 5C). The relative expression levels of CDS and UTR regions showed no statistically significant difference (*p* > 0.05) in small follicular fluid samples (Figure 5D). For other transcripts, the 5′UTR regions were significantly higher (*p* < 0.05) in large follicular fluid than CDS and 3′UTR regions (Figure 5E and Supplementary Figure S4A). Moreover, the result showed no significant difference in the relative expression of the UTR and CDS regions of the other transcripts in small follicular fluid samples (Figure 5F and Supplementary Figure S4B). Furthermore, for full-length transcripts, the CDS and UTR regions of large follicular fluid were remarkably lower than those of small follicular fluid samples (*p* < 0.05) (Figure 5G). For other transcripts, the CDS and UTR regions of large follicular fluid were significantly lower than those of small follicular fluid (*p* < 0.05) (Figure 5H and Supplementary Figure S4C,D,E).

As shown in Figures 5G,I, this result indicated that about 17.13 % and 35.75% of spliced junctions were derived from known RNAs in large follicular fluid and small follicular fluid samples, respectively. Furthermore, 81.69 % and 62.63% of spliced junctions were derived from novel junctions in large follicular fluid and small follicular fluid samples (Figures 5G,I)**,** respectively. Also, the known RNA was remarkably higher (*p* < 0.05) in small follicular fluid than in large follicular fluid, whereas the opposite was found for novel junctions (Figure 5K).

### Presence of cell-free RNAs derived from tissue-specific genes in human follicular fluid

**FIGURE 6:**
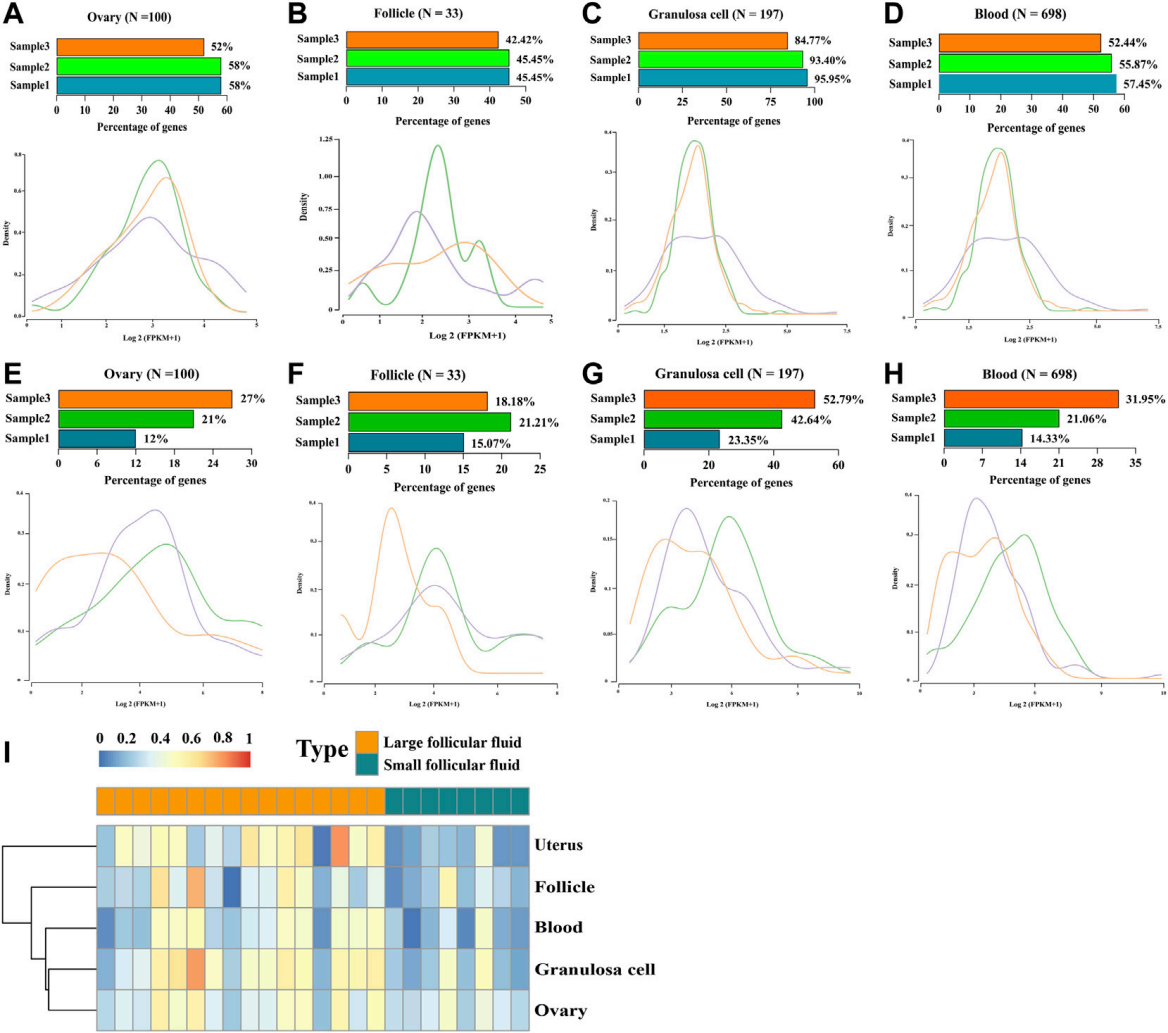
Origin of tissue-specific genes in follicular fluid. **(A–D)** Number and expression levels of the RNA derived from **(A)** ovary-, **(B)** follicle-, **(C)** granulosa cell-, and **(D)** blood-specific genes in large follicular fluid. (Upper) Percentage of RNA detected in each sample. N, the total number of genes specifically expressed in this tissue. (Lower) Distribution of RNA expression from the corresponding tissue-specific genes. **(E–H)** Number and expression levels of the RNA derived from **(E)** ovary-, **(F)** follicle-, **(G)** granulosa cell-, and **(H)** blood-specific genes in small follicular fluid. (Upper) percentage of RNA detected in each sample. N, the total number of genes specifically expressed in this tissue. (Lower) distribution of RNA expression from the corresponding tissue-specific genes. **(I)** Scores of four tissue-specific genes per follicular fluid sample were analyzed by using the single-sample Gene Set Enrichment Analysis (ssGSEA) scores (red shows a high score, and blue shows a low score).

We examined whether tissue-specific gene expression contributed to follicular fluid RNA in circulation. For this purpose, we used reported genes with tissue-specific expressions, including 100, 32, 197, and 698 genes specifically expressed in the ovary, follicle, granulosa cells, and blood, respectively. The result showed that follicular fluid cfRNAs from all tissue-specific genes were detected in all three samples (Figures 6A–H, upper). Furthermore, the expression levels, as measured by FPKM, were not concentrated near 0 (Figures 6A–H, lower). In contrast, the RNA abundances (FPKM) of tissue-specific genes showed unimodal distributions and positive patterns. The single-sample Gene Set Enrichment Analysis (ssGSEA) score analysis showed that large follicular fluid tissue-specific gene scores were higher than those of small follicular fluid (Figure 6I).

### Follicular fluid RNA can be used as a potential indicator to distinguish the quality of follicles

**FIGURE 7:**
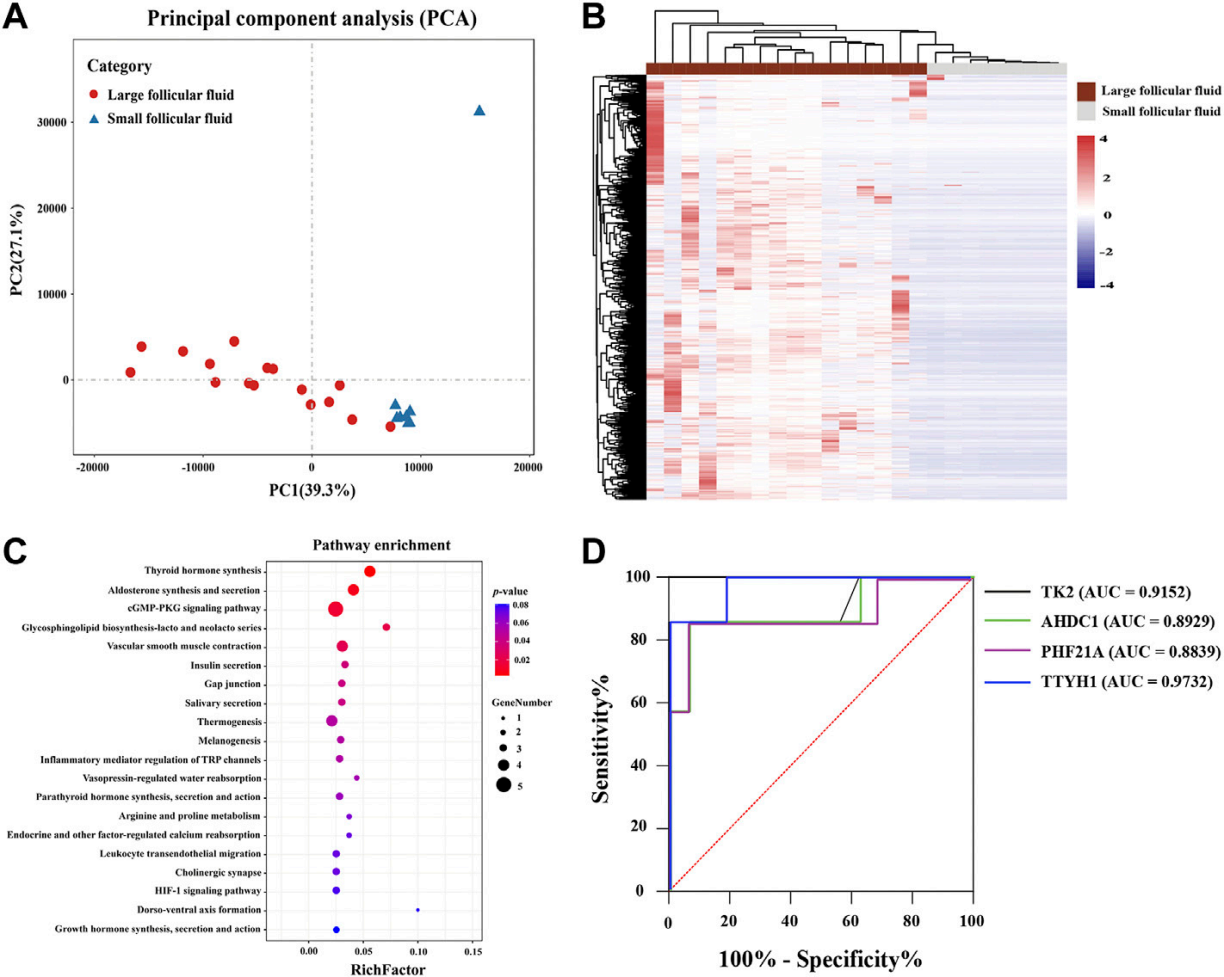
**(A)** PCA plot shown follicular fluid cell-free RNA expression pattern per sample. **(B)** Heatmap of differentially expressed RNAs between large follicular fluid and small follicular fluid. Each column represents a separate sample, and each row represents a gene. Red indicates high expression, and purple indicates low expression; KEGG pathway enrichment analysis for differentially expressed RNAs is shown in **(C)**, which was conducted using DAVID. **(D)** ROC curves showed the analysis of four cell-free RNAs in predicting the different-sized follicles’ follicular fluid. Black, green, red, and blue represent TK2, AHDC1, PHF21A, and TTYH1, respectively. Red line represents the reference line.

As shown in Figure 7A, PCA showed that the cfRNA maps of large follicular fluid usually differed from those of small follicular fluid. A total of 410 differentially expression cfRNAs were identified in large follicular fluid samples compared with small follicular fluid samples by the Mann–Whitney U test (FDR < 0.01, fold change > 2). Unsupervised hierarchical clustering revealed a clear separation of large follicular fluid and small follicular fluid (Figure 7B). KEGG pathway analysis revealed that differentially expressed cfRNAs were enriched for some pathways, such as thyroid hormone synthesis, the cGMP-PKG signaling pathway, and inflammatory mediator regulation of TRP channels (Figure 7C).

In addition, only upregulated cfRNAs (n = 409) in follicular fluid were considered (FDR < 0.01, fold change > 2). The random forest algorithm was used to analyze the selected cell-free RNA markers to reduce the number of variables. Finally, four cfRNA markers (TK2, AHDC1, PHF21A, and TTYH1) were selected. To evaluate the feasibility of using the selected four RNAs in follicular fluid as a difference between different follicular fluid sizes, we analyzed the sensitivity and specificity of these RNAs. As is presented in Figure 7D, the ROC curve indicated a great potential in predicting the follicular fluid of follicles of different sizes (AUC >0.88).

## Discussion

To our knowledge, this study is the first to assess the transcriptome range of cfRNA in follicular fluid. Previous work mainly focused on circulating miRNAs (Hossain et al., 2012; Xiao et al., 2014), which is difficult to explain due to its pleiotropic effect on gene regulation, and rarely translated into clinical practice. In this study, we analyzed the characteristics of follicular fluid cfRNAs, and their existence form, regularity, and the potential to predict different follicle sizes were further studied. The results found that the concentration of follicular fluid cfRNAs ranged from 0.78 ng/ml to 8.76 ng/ml, and the fragment length was from 20 to 200 nucleotides. For the abundance and species of follicular fluid cfRNA transcripts, this result showed that the follicular fluid cfRNAs could be divided into 37 Ensembl RNA biotypes. A large number of mRNAs, circRNAs, and lncRNAs were observed in follicular fluid, and the amount of large follicular fluid was remarkably higher than that of small follicular fluid. The integrity of mRNA analysis showed that follicular fluid contained a large amount of intact mRNA and splice junctions. Follicular fluid may contain a large number of tissue-derived genes, and the supply and demand of tissue-derived cfRNAs in follicular fluid is in a balanced state. KEGG pathway analysis found that differentially expression cfRNAs were recruited in some pathways, such as thyroid hormone synthesis, the cGMP-PKG signaling pathway, and inflammatory mediator regulation of TRP channels. In addition, our results further showed that four cfRNAs can serve as a potential indicator to predict follicles of different sizes. The ROC curve showed great potential in predicting the different follicle sizes [area under the curve (AUC) > 0.88].

In this study, the biological variation of cfRNA content in different follicular fluid samples may be due to the fact that different follicles have different abilities to release RNAs into the follicular fluid. In addition, this study showed that the fragment length of large follicular fluid cfRNAs was higher than that of small follicular fluid, but there was no significant difference. This was supported by the fact that in mammals, including humans, the concentration of magnesium ions in small follicular fluid was higher than that of large follicular fluid, which might lead to RNA degradation (Nandi et al., 2007; Malizia et al., 2010; Nishigaki et al., 2011). Furthermore, as in the development of the follicle, the liquid environment of the follicle changes from alkaline to acidic under the influence of phosphatase to alleviate the degradation of RNAs, which explains the scattered distribution of RNA fragments in small follicular fluid samples (Nandi, Kumar, Manjunatha and Gupta, 2007; Nishigaki, Okada, Okamoto, Sugiyama, Miyazaki, Yasuda and Kanzaki, 2011).

Furthermore, this result indicated that between-sample correlations were always lower than within-sample correlations. The low correlation may be due to the fact that the complexity of the follicular fluid composition results in great variation between samples. Additionally, the degree of development of different follicles can also lead to great differences between samples. Moreover, this study found that the sizes of follicular fluid cfRNA sequenced fragments were about 200 bp, and the maximum read fragment length was 173361750. This suggests that the follicular fluid may contain intact fragments of no degraded RNAs. In addition, we compared the correlations between follicular fluids of different sizes. This result indicated that the correlation between large follicular fluid samples was significantly higher than that between small follicular fluid samples. This may be due to the fact that the composition of the follicular fluid is highly variable and closely related to the stage of follicular development. For mature large follicular fluid, its components are relatively stable (Pla et al., 2021). However, small follicular fluid stops developing due to many unknown reasons, resulting in the complexity and diversity of components. Additionally, the distribution of read fragments in large follicular fluid was almost concentrated at about 200 bp, but the distribution of read fragments in small follicular fluid was diffuse and irregular. Although there was no remarkable difference between large follicular fluid and small follicular fluid samples, this was consistent with Agilent analysis. This can be supported by the fact that the RNA degradation degree of small follicular fluid was more severe than that of large follicular fluid. This might inevitably lead to the dispersed distribution of read fragments.

Most previous analyses focused on miRNAs (Sang et al., 2013). However, most sequencing reads could not be aligned to miRNAs, which raises the question of what other RNAs are present in the human follicular fluid. This study revealed a large number of long RNA fragments in the human follicular fluid. These fragments are usually 200 nt. They were derived from protein_coding RNAs, lncRNAs, small RNAs, and other ncRNAs. Also, these RNAs are highly expressed in follicular fluid. This demonstrated that follicular fluid contained other types of RNAs besides miRNA, which provided a certain reference for the future study of follicular fluid. Additionally, we also found that many different RNA biotypes were evenly distributed in large follicular fluid and small follicular fluid samples, and many transcripts were detected in both large follicular fluid and small follicular fluid samples. These data demonstrate the effectiveness of our sequencing method and the relative consistency between large groups of follicular fluid in the RNA profiles. In both large follicular fluid and small follicular fluid samples, the protein_coding RNAs were more abundant than those of lncRNAs, small RNAs, and other ncRNAs in total aligned reads. These data are consistent with a recent report comparing RNA extracted from extracellular vesicles (EVs) in human blood (Li Y et al., 2019). In addition, for the distribution of genes, we found that the proportion of protein-coding genes in small follicular fluid was remarkably higher than that of large follicular fluid. Previous studies have shown that the protein_coding genes of parental origin will be degraded with the development of the embryo (Wang Y et al., 2021). Hence, we assume that the protein_coding genes may gradually degrade in the process of oocyte development, resulting in lower protein_coding genes in large follicular fluid than in small follicular fluid.

In this study, we found that most follicular fluid mRNAs were complete fragments (full-length coverage of protein_coding genes >83%). This result was different from previous studies, in which the RNAs of body fluids are fragments (Batagov and Kurochkin, 2013; Wei et al., 2017). Therefore, we infer that follicular fluid may contain a large number of microvesicles to prevent the degradation of RNAs (Li X et al., 2019). This also explains why there are a large number of intact mRNAs in follicular fluid, which provides a reference for subsequent studies. In addition, this study found that 5′UTR regions were remarkably enriched in large follicular fluid compared to CDS regions. It should be noted that the cellular RNA content in large follicular fluid was very different from that in plasma, suggesting that this feature might be limited to large follicular fluid. For small follicular fluid, the expression levels of CDS and UTR regions were significantly higher than those of large follicular fluid. This result may be reflected in the higher transcriptional activity of genes in small follicular fluid than in large follicle fluid.

This study found that most tissue-specific RNAs, including the ovary, follicle, granulosa cells, and blood-specific RNAs, were detected in the human follicular fluid. The proportion of tissue-specific genes in large follicular fluid was remarkably higher than that of small follicular fluid. This result might be attributed to the release of various metabolites and molecules into the follicular fluid by tissues during follicular development, thus promoting follicular development. Furthermore, this study demonstrated that the proportion of tissue-specific genes from granulosa cells in the follicular fluid was significantly higher than that of tissues from other sources. This result can interpret that the proportion of granulosa cells in follicular fluid was significantly higher than that in other types of cells. This also proves that the contamination of granulosa cell source is the bottleneck problem to improve the detection of cfRNAs in the follicular fluid. Moreover, the RNA abundances (FPKM) of tissue-specific genes indicated unimodal distributions and positive patterns. These distributions show that tissue-derived cfRNAs are at a balance supply and clearance in follicular fluid.

PCA results indicated that the genes of different-sized follicles’ follicular fluid could be completely separated, which suggested that the follicular fluid of different-sized follicles could be distinguished by using some genes. Moreover, we performed unsupervised clustering of differentially expressed genes and found that the follicular fluid of follicles of different sizes could be clearly separated. This again supported that cfRNAs could be used to predict different-sized follicles’ follicular fluid. Previous studies have shown that cyclic guanosine monophosphate (cGMP) and cGMP-dependent protein kinase G (PKG) were expressed in neonatal ovaries of pre-granulosa cells, promoting primordial follicle activation, oocyte growth, and granulosa cell proliferation (Tian et al., 2018; Zhao et al., 2020). This study found that the cGMP-PKG signaling pathway was significantly enriched, which indicated that the activation of the cGMP-PKG signaling pathway may be closely related to the development of follicles. Furthermore, we screened four upregulated genes by using a random forest algorithm, including TK2, AHDC1, PHF21A, and TTYH1, which are closely related to follicular fluid sizes. The ROC curve showed great potential in predicting the different follicle sizes [area under the curve (AUC) > 0.88]. Furthermore, studies showed that these transcripts were widely distributed at cleavage, blastocyst, and gastrula stages and could promote early embryo development (Kumada et al., 2010; Kim et al., 2012). Overall, these findings indicated that cfRNAs in the earlier microenvironment of follicles could be used as a potential non-invasive biomarker to predict subsequent follicle sizes, as an alternative or supplemental method of the currently applied morphological criteria.

## Conclusion

In conclusion, our study revealed that a large number of cfRNAs could be detected in follicular fluid and could serve as a potential non-invasive biomarker in distinguishing between follicles of different sizes. These results may inform the study of the utility and implementation of cfRNAs in clinical practice.

## Data Availability

The data presented in the study are deposited in the NCBI repository, accession number PRJNA879096.

## References

[B1] Batagov A. O. Kurochkin I. V. (2013). Exosomes secreted by human cells transport largely mRNA fragments that are enriched in the 3'-untranslated regions. Biol. Direct 8, 12. 10.1186/1745-6150-8-12 23758897 PMC3732077

[B2] Berker B. Kaya C. Aytac R. Satiroglu H. (2009). Homocysteine concentrations in follicular fluid are associated with poor oocyte and embryo qualities in polycystic ovary syndrome patients undergoing assisted reproduction. Hum. Reprod. 24, 2293–2302. 10.1093/humrep/dep069 19443458

[B3] Boivin J. Bunting L. Collins J. A. Nygren K. G. (2007). International estimates of infertility prevalence and treatment-seeking: Potential need and demand for infertility medical care. Hum. Reprod. 22, 1506–1512. 10.1093/humrep/dem046 17376819

[B4] Braga D. P. Setti A. S. Lo Turco E. G. Cordeiro F. B. Cabral E. C. Cortezzi S. S. (2016). Protein expression in human cumulus cells as an indicator of blastocyst formation and pregnancy success. J. Assist. Reprod. Genet. 33, 1571–1583. 10.1007/s10815-016-0800-7 27614633 PMC5171892

[B5] Cai Y. Y. Lin N. Zhong L. P. Duan H. J. Dong Y. H. Wu Z. (2019). Serum and follicular fluid thyroid hormone levels and assisted reproductive technology outcomes. Reprod. Biol. Endocrinol. 17, 90. 10.1186/s12958-019-0529-0 31699106 PMC6839061

[B6] Da Broi M. G. Giorgi V. S. I. Wang F. Keefe D. L. Albertini D. Navarro P. A. (2018). Influence of follicular fluid and cumulus cells on oocyte quality: Clinical implications. J. Assist. Reprod. Genet. 35, 735–751. 10.1007/s10815-018-1143-3 29497954 PMC5984887

[B7] De Geyter C. (2019). Assisted reproductive technology: Impact on society and need for surveillance. Best. Pract. Res. Clin. Endocrinol. Metab. 33, 3–8. 10.1016/j.beem.2019.01.004 30799230

[B8] De Rycke M. Goossens V. Kokkali G. Meijer-Hoogeveen M. Coonen E. Moutou C. (2017). ESHRE PGD consortium data collection XIV-XV: Cycles from january 2011 to december 2012 with pregnancy follow-up to october 2013. Hum. Reprod. 32, 1974–1994. 10.1093/humrep/dex265 29117384

[B9] Fu J. Qu R. G. Zhang Y. J. Gu R. H. Li X. Sun Y. J. (2018). Screening of miRNAs in human follicular fluid reveals an inverse relationship between microRNA-663b expression and blastocyst formation. Reprod. Biomed. Online 37, 25–32. 10.1016/j.rbmo.2018.03.021 29703434

[B10] Gao Y. Wang J. Zhao F. (2015). Ciri: An efficient and unbiased algorithm for de novo circular RNA identification. Genome Biol. 16, 4. 10.1186/s13059-014-0571-3 25583365 PMC4316645

[B11] Hanrieder J. Nyakas A. Naessén T. Bergquist J. (2008). Proteomic analysis of human follicular fluid using an alternative bottom-up approach. J. Proteome Res. 7, 443–449. 10.1021/pr070277z 18047273

[B12] Hanrieder J. Zuberovic A. Bergquist J. (2009). Surface modified capillary electrophoresis combined with in solution isoelectric focusing and MALDI-TOF/TOF MS: A gel-free multidimensional electrophoresis approach for proteomic profiling--exemplified on human follicular fluid. J. Chromatogr. A 1216, 3621–3628. 10.1016/j.chroma.2008.12.026 19155017

[B13] Hossain M. M. Salilew-Wondim D. Schellander K. Tesfaye D. (2012). The role of microRNAs in mammalian oocytes and embryos. Anim. Reprod. Sci. 134, 36–44. 10.1016/j.anireprosci.2012.08.009 22921265

[B14] Huang X. Hong L. Wu Y. Chen M. Kong P. Ruan J. (2021). Raman spectrum of follicular fluid: A potential biomarker for oocyte developmental competence in polycystic ovary syndrome. Front. Cell Dev. Biol. 9, 777224. 10.3389/fcell.2021.777224 34858993 PMC8632455

[B15] Huo P. Zhang N. Zhang P. Wu X. (2020). The levels of follicular fluid cell-free mitochondrial DNA could serve as a biomarker for pregnancy success in patients with small ovarian endometriosis cysts: A case-control study. Med. Baltim. 99, e23348. 10.1097/MD.0000000000023348 PMC7710174 33235102

[B16] Inhorn M. C. Patrizio P. (2015). Infertility around the globe: New thinking on gender, reproductive technologies and global movements in the 21st century. Hum. Reprod. Update 21, 411–426. 10.1093/humupd/dmv016 25801630

[B17] Jiao J. Shi B. Wang T. Fang Y. Cao T. Zhou Y. (2018). Characterization of long non-coding RNA and messenger RNA profiles in follicular fluid from mature and immature ovarian follicles of healthy women and women with polycystic ovary syndrome. Hum. Reprod. 33, 1735–1748. 10.1093/humrep/dey255 30052945

[B18] Kim D. Langmead B. Salzberg S. L. (2015). Hisat: A fast spliced aligner with low memory requirements. Nat. Methods 12, 357–360. 10.1038/nmeth.3317 25751142 PMC4655817

[B19] Kim H. G. Kim H. T. Leach N. T. Lan F. Ullmann R. Silahtaroglu A. (2012). Translocations disrupting PHF21A in the Potocki-Shaffer-syndrome region are associated with intellectual disability and craniofacial anomalies. Am. J. Hum. Genet. 91, 56–72. 10.1016/j.ajhg.2012.05.005 22770980 PMC3397276

[B20] Kim Y. S. Kim M. S. Lee S. H. Choi B. C. Lim J. M. Cha K. Y. (2006). Proteomic analysis of recurrent spontaneous abortion: Identification of an inadequately expressed set of proteins in human follicular fluid. Proteomics 6, 3445–3454. 10.1002/pmic.200500775 16637005

[B21] Kumada T. Yamanaka Y. Kitano A. Shibata M. Awaya T. Kato T. (2010). Ttyh1, a Ca(2+)-binding protein localized to the endoplasmic reticulum, is required for early embryonic development. Dev. Dyn. 239, 2233–2245. 10.1002/dvdy.22348 20568244

[B22] Kushnir V. A. Barad D. H. Albertini D. F. Darmon S. K. Gleicher N. (2017). Systematic review of worldwide trends in assisted reproductive technology 2004-2013. Reprod. Biol. Endocrinol. 15, 6. 10.1186/s12958-016-0225-2 28069012 PMC5223447

[B23] Lazzarino G. Pallisco R. Bilotta G. Listorti I. Mangione R. Saab M. W. (2021). Altered follicular fluid metabolic pattern correlates with female infertility and outcome measures of *in vitro* fertilization. Int. J. Mol. Sci. 22, 8735. 10.3390/ijms22168735 34445441 PMC8395780

[B24] Li H. Durbin R. (2009). Fast and accurate short read alignment with Burrows–Wheeler transform. bioinformatics 25, 1754–1760. 10.1093/bioinformatics/btp324 19451168 PMC2705234

[B25] Li X X. Zhang W. Fu J. Xu Y. Gu R. Qu R. (2019). MicroRNA-451 is downregulated in the follicular fluid of women with endometriosis and influences mouse and human embryonic potential. Reprod. Biol. Endocrinol. 17, 96. 10.1186/s12958-019-0538-z 31744497 PMC6862852

[B26] Li Y Y. Zhao J. Yu S. Wang Z. He X. Su Y. (2019). Extracellular vesicles long RNA sequencing reveals abundant mRNA, circRNA, and lncRNA in human blood as potential biomarkers for cancer diagnosis. Clin. Chem. 65, 798–808. 10.1373/clinchem.2018.301291 30914410

[B27] Malizia B. A. Wook Y. S. Penzias A. S. Usheva A. (2010). The human ovarian follicular fluid level of interleukin-8 is associated with follicular size and patient age. Fertil. Steril. 93, 537–543. 10.1016/j.fertnstert.2008.11.033 19285667

[B28] Montani D. A. Braga D. Borges E. Jr. Camargo M. Cordeiro F. B. Pilau E. J. (2019). Understanding mechanisms of oocyte development by follicular fluid lipidomics. J. Assist. Reprod. Genet. 36, 1003–1011. 10.1007/s10815-019-01428-7 31011990 PMC6541691

[B29] Nandi S. Kumar V. G. Manjunatha B. M. Gupta P. S. (2007). Biochemical composition of ovine follicular fluid in relation to follicle size. Dev. Growth Differ. 49, 61–66. 10.1111/j.1440-169X.2007.00901.x 17227345

[B30] Nishigaki A. Okada H. Okamoto R. Sugiyama S. Miyazaki K. Yasuda K. (2011). Concentrations of stromal cell-derived factor-1 and vascular endothelial growth factor in relation to the diameter of human follicles. Fertil. Steril. 95, 742–746. 10.1016/j.fertnstert.2010.10.028 21071025

[B31] Patrizio P. Fragouli E. Bianchi V. Borini A. Wells D. (2007). Molecular methods for selection of the ideal oocyte. Reprod. Biomed. Online 15, 346–353. 10.1016/s1472-6483(10)60349-5 17854537

[B32] Pla I. Sanchez A. Pors S. E. Pawlowski K. Appelqvist R. Sahlin K. B. (2021). Proteome of fluid from human ovarian small antral follicles reveals insights in folliculogenesis and oocyte maturation. Hum. Reprod. 36, 756–770. 10.1093/humrep/deaa335 33313811 PMC7891813

[B33] Qasemi M. Amidi F. (2020). Extracellular microRNA profiling in human follicular fluid: New biomarkers in female reproductive potential. J. Assist. Reprod. Genet. 37, 1769–1780. 10.1007/s10815-020-01860-0 32642870 PMC7468023

[B34] Sang Q. Yao Z. Wang H. Feng R. Wang H. Zhao X. (2013). Identification of microRNAs in human follicular fluid: Characterization of microRNAs that govern steroidogenesis *in vitro* and are associated with polycystic ovary syndrome *in vivo* . J. Clin. Endocrinol. Metab. 98, 3068–3079. 10.1210/jc.2013-1715 23666971

[B35] Santonocito M. Vento M. Guglielmino M. R. Battaglia R. Wahlgren J. Ragusa M. (2014). Molecular characterization of exosomes and their microRNA cargo in human follicular fluid: Bioinformatic analysis reveals that exosomal microRNAs control pathways involved in follicular maturation. Fertil. Steril. 102, 1751–1761. e1751. 10.1016/j.fertnstert.2014.08.005 25241362

[B36] Scalici E. Gala A. Ferrieres A. Vincens C. Hamamah S. (2014). Does embryo morphology constitute a reliable criterion for embryo selection? Gynecol. Obstet. Fertil. 42, 661–664. 10.1016/j.gyobfe.2014.07.036 25153441

[B37] Tian Y. Heng D. Xu K. Liu W. Weng X. Hu X. (2018). cGMP/PKG-I pathway-mediated GLUT1/4 regulation by NO in female rat granulosa cells. Endocrinology 159, 1147–1158. 10.1210/en.2017-00863 29300939

[B38] Wang J J. Zheng W. Zhang S. Yan K. Jin M. Hu H. (2021). Publisher Correction to: An increase of phosphatidylcholines in follicular fluid implies attenuation of embryo quality on day 3 post-fertilization. BMC Biol. 19, 234. 10.1186/s12915-021-01175-1 34727924 PMC8564983

[B39] Wang Q. Sun Q.-Y. (2006). Evaluation of oocyte quality: Morphological, cellular and molecular predictors. Reprod. Fertil. Dev. 19, 1–12. 10.1071/rd06103 17389130

[B40] Wang Y Y. Yuan P. Yan Z. Yang M. Huo Y. Nie Y. (2021). Single-cell multiomics sequencing reveals the functional regulatory landscape of early embryos. Nat. Commun. 12, 1247. 10.1038/s41467-021-21409-8 33623021 PMC7902657

[B41] Wei Z. Batagov A. O. Schinelli S. Wang J. Wang Y. El Fatimy R. (2017). Coding and noncoding landscape of extracellular RNA released by human glioma stem cells. Nat. Commun. 8, 1145. 10.1038/s41467-017-01196-x 29074968 PMC5658400

[B42] Wu Y. T. Tang L. Cai J. Lu X. E. Xu J. Zhu X. M. (2007). High bone morphogenetic protein-15 level in follicular fluid is associated with high quality oocyte and subsequent embryonic development. Hum. Reprod. 22, 1526–1531. 10.1093/humrep/dem029 17347167

[B43] Xiao G. Xia C. Yang J. Liu J. Du H. Kang X. (2014). MiR-133b regulates the expression of the actin protein TAGLN2 during oocyte growth and maturation: A potential target for infertility therapy. PLoS One 9, e100751. 10.1371/journal.pone.0100751 24959893 PMC4069098

[B44] Yang X. Li Y. Li C. Zhang W. (2014). Current overview of pregnancy complications and live-birth outcome of assisted reproductive technology in mainland China. Fertil. Steril. 101, 385–391. 10.1016/j.fertnstert.2013.10.017 24269043

[B45] Zhang D. Lv J. Tang R. Feng Y. Zhao Y. Fei X. (2021). Association of exosomal microRNAs in human ovarian follicular fluid with oocyte quality. Biochem. Biophys. Res. Commun. 534, 468–473. 10.1016/j.bbrc.2020.11.058 33256978

[B46] Zhao P. Song Z. Wang Y. Cai H. Du X. Li C. (2020). The endothelial nitric oxide synthase/cyclic guanosine monophosphate/protein kinase G pathway activates primordial follicles. Aging (Albany NY) 13, 1096–1119. 10.18632/aging.202235 33291075 PMC7835019

