# Supplementary figures and images for: Extracellular cell-free RNA profile in human large follicles and small follicles
Source: Front Cell Dev Biol. 2022 Sep 26;10:940336. doi: 10.3389/fcell.2022.940336 (PMC9549077; doi:10.3389/fcell.2022.940336)

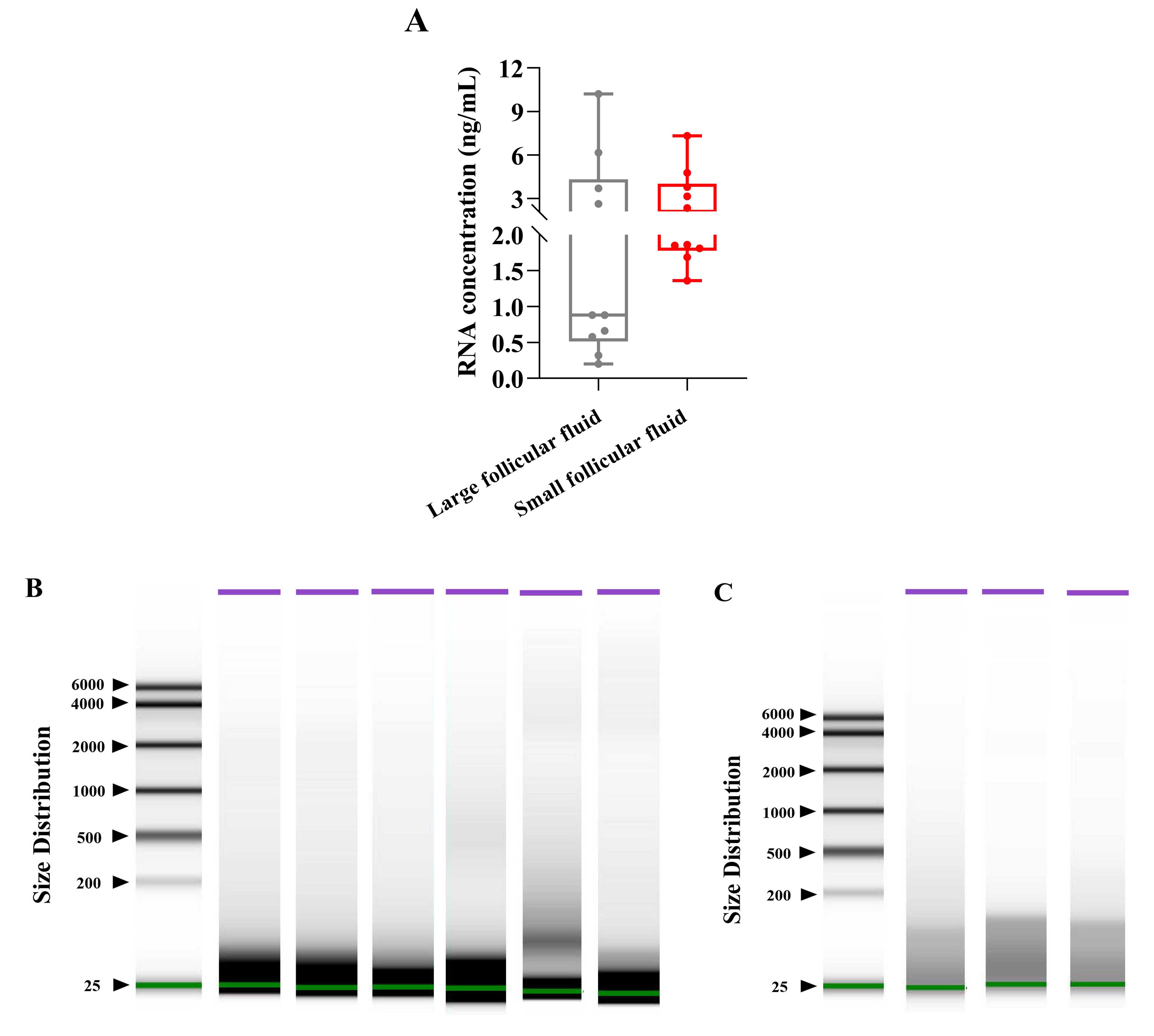

Supplement: Supplementary file 2 [file Image1.JPEG]
