# Supplementary material for: Extracellular cell-free RNA profile in human large follicles and small follicles
Source: Front Cell Dev Biol. 2022 Sep 26;10:940336. doi: 10.3389/fcell.2022.940336 (PMC9549077; doi:10.3389/fcell.2022.940336)
Supplement: Supplementary file 5 [file Table1.doc]

**Table S1** Patients’ characteristics of the study population (*n* = 20).

| Variable | Mean | n (%) | Min-max | SEM |
| --- | --- | --- | --- | --- |
| Age (years) | 32.27 |  | 27~43 | 5.78 |
| < 36 years | - | 17 (85.00) | - | - |
| > 36 years | - | 3 (15.00) | - | - |
| IVF | - | 9 (45.00) | - | - |
| ICSI | - | 11 (55.00) | - | - |
| BMI (kg/m^2^) | 22.1 | - | 18~25.4 | 2.29 |
| FSH (pmol/L) | 7.21 | - | 7.05~10.65 | 1.63 |
| LH (pmol/L) | 3.88 | - | 0.06~8.38 | 2.38 |
| 17β estradiol (E2;pmol/L) | 134.97 | - | 51.5~257.2 | 50.97 |
| AMH* (ng/mL) | 4.72 | - | 1.03~10.33 | 3.36 |
| Antral follicle count | 16 | - | 1~26 | 7.41 |
| Normal follicle count | 12.2 | - | 5~21 | 5.27 |
| Normal ovarian reserve (%) | - | 13 (80.9) | - | - |
| Peak E2 level (pmol/L)* | 16174.63 | - | 5014.9~39742.2 | 10491.06 |

AMH: anti-Mullerian hormone

Peak E2 level: E2 level measured at the time of the injection of hCG
